# Professor Volker Hömberg, President of the World Federation for NeuroRehabilitation: Adapted Interview from the 12^th^ World Congress for NeuroRehabilitation (WCNR) – Vienna, 2022

**DOI:** 10.25122/jml-2023-1002

**Published:** 2023-01

**Authors:** Alexandra Gherman, Stefana-Andrada Dobran

**Affiliations:** 1 RoNeuro Institute for Neurological Research and Diagnostic, Cluj-Napoca, Romania; 2 Sociology Department, Babes-Bolyai University, Cluj-Napoca, Romania

**Interviewer: Alexandra Gherman**

**Interviewee: Professor Volker Hömberg**

Prof. Hömberg had his medical education at the Universities of Düsseldorf, Freiburg, and Boston Massachusetts. After taking electives in Neurology at Boston City Hospital and the National Hospital for Nervous Diseases Queens Square London, he was a research fellow at the C. and O. Vogt Institute for Brain Research in Düsseldorf. He was involved in the setup of many in- and outpatient rehabilitation hospitals in Germany. In 2001 he started the St. Mauritius Therapy Clinic in Meerbusch near Düsseldorf, and since 2011 he has been the Director of the Department of Neurology at the Gesundheitszentrum Bad Wimpfen and worked as senior neurology group leader for the SRH-Group, one of the biggest hospital groups in Germany. He was the founder, president, and vice president of the German Society for Neurorehabilitation for many years. He served as Secretary General for the World Federation of Neurorehabilitation (WFNR) for more than 12 years and is Vice President of the European Federation of Neurorehabilitation Societies (EFNR). He is a regular reviewer and co-editor for many international peer-reviewing journals. He is a regular (co-) programme chairman for neurorehabilitation for major international meetings such as the World- and European Neurorehabilitation Congresses (WCNR, ECNR), Controversies in Neurology (CONy) and the European Stroke Congress (ESC).

He has published more than 250 articles in international peer-reviewed journals and many book chapters. His primary scientific interests are the fields of motor rehabilitation, cognition epistemology, neurological music therapy, and pharmacology in neurorehabilitation.

**A.G.:** Hello, Professor Hömberg! We are here in Vienna for the 12^th^ World Congress for NeuroRehabilitation, organised by the World Federation for NeuroRehabilitation. What is your first-hand opinion of the event so far?

**V.H.:** Well, I am very happy. We have a very big meeting in terms of the scientific content, we have a very big meeting in terms of attendance – 1.400 and more people, which is nice, which is also a reflection of these pandemic times – people are pretty happy to meet, to be able to meet in person, person-to-person again. And enjoy also some extracurricular activities at these meetings, of course.

**A.G.:** Thank you very much! What is the overarching theme of this year's congress?

**V.H.:** Well, usually, at the world congresses, we try to cover a very wide span both in the scientific and educational fields. I think an important coming up problem, for all of us, for years, is the use of neurotechnologies to be embedded into rehabilitation. And that is a fair bit of what we are discussing here, [it] is related to this topic. The other major topic, of course, is communication tools, and that will also in [the] future be a very important aspect of our future work for the Federation, as well. And during my coming presidency now, I think it will be one of my main topics. We must consider that hundreds of millions of people in the world who do not have any access to any form of rehabilitation and certainly not in the sense we know it from Western Europe, from North America, and from parts of Asia. So, we have to make our minds up about what we can do to reach those people in a low-threshold and affordable form. This is only possible by strengthening our efforts in education, training, coaching, and teaching patients, caregivers, relatives in the communities.

**A.G.:** Thank you very much! From your perspective, Professor Hömberg, what is the role of hybrid multidisciplinary events in developing neurorehabilitation research and practice, and which similar avenues are worth exploring?

**V.H.:** Well, we have started with the hybrid formats during the pandemic, of course. During that time, we have learned that it is a very useful tool for the distribution of information, similar to what we did by person-to-person congresses before. In the future, I think that we would probably stay with elements of a hybrid form also for other meetings. "Hybrid" has several advantages. One advantage is, of course, that people from remote areas can avoid travel and accommodation costs to join meetings. Another advantage is that, in the hybrid form, you can easily store the content and make it available also on demand. Nevertheless, I think that it's nice that we can have person-to-person meetings again, considering all the positive important psychological advantages of meeting in person.

**A.G.:** As the incoming President of the World Federation for NeuroRehabilitation, what do you consider to be the most important recent advances in the field of neurorehabilitation?

**V.H.:** Well, there are a lot of advances which have been made over the last two decades. However, progress usually is slow. And, of course, we have to think – "*Do we really have game-changers so far?*" And I think we don't have "**the**" game changer at the moment. I mentioned before that the use of digital technologies for educational activities will be a very important aspect. What could also be another important aspect is a change in our thinking about how to derive knowledge from data, *e.g*., generate knowledge from trials. And we certainly have to think about running more informative and better trials. Throughout the world, every month, many (up to 50–100) good papers appear in many well-edited journals around the world. This is nice, not only in the sense that sort of a "PhD factory" is working, but also showing that neurorehab is a dynamic field. Many of these studies, although they are of good or even superb scientific quality, their relevance to the field is nevertheless low because the original question for the research done was really not pertinent to what we actually need. Another problem is guidelines. Guidelines certainly are useful; guidelines are important. We have a problem with the adherence to guidelines. And that is also a project for the future. We are now starting to do better informative surveys on this for stroke, traumatic brain injury, and cerebral palsy in many parts of the world.

**A.G.:** Thank you very much for your answer! What can be done, at an international scale, to strengthen research and collaboration in neurorehabilitation?

**V.H.:** Well, we certainly need a strengthening of research and academic activities in this field. There are many aspects to that. One is basic and clinical science – conducting trials, running experiments, and increasing the scientific content in neurorehabilitation. We have always conceived, and this is part of the DNA of WFNR - to conceive neurorehabilitation as applied neuroscience. We are lacking, in many parts of the world, academic institutions in neurorehabilitation or rehabilitation in general. We certainly need strengthening of the academic impact of neurorehabilitation worldwide. And we also, of course, in the future, need to have more multicentre, international, cooperative trials. This has to be accompanied by new ways of academic education using digital technologies and also artificial intelligence for a rapid updating of knowledge needed to make clinical decisions and marshal individual patients' rehabilitation plans.

**A.G.:** Yes, thank you very much! How can we address disparities in rehabilitation for low- and middle-income countries?

**V.H.:** Yes, this is a tough issue; I mentioned that already. What I expect within the next couple of years – and I would certainly put a major emphasis on this, is to use digital communication technologies to reach more people in affordable and, let's say, really low-threshold form. And if there was anything good to learn during this pandemic, it was that we have learned to use digital communication tools more easily and efficiently. One of our ongoing WFNR projects is to create a metaverse rehab hospital, a virtual rehabilitation hospital with all the components [of] a classical, let's say, European or American rehabilitation hospital. In this form, it can become easily available to people everywhere. They just need to have a smartphone or a tablet and an internet connection. And this is available almost everywhere now.

**A.G.:** Yes.

**V.H.:** It's easy, it's relatively cheap, and by doing so, we can reach a much, much higher number of patients and relatives, help them make decisions, and also training them skills and procedures. We certainly have to emphasize more training for professional and especially also for non-professional (*e.g*., care-givers) people dealing with patients in rehab. of professional health people in many countries. That is a long-lasting process, and that is also a process in which we have to think about how we could improve the didactics to do that and how we can reach more people. Many countries don't have a single physiotherapist there for instance, to treat a patient. And that, of course, has to be changed. A component of this is that we will have a major emphasis on training non-professionals.

**A.G.:** Thank you! What is the impact of mentorship programs on the new generations of neurorehabilitation specialists?

**V.H.:** Yes, I think that is a very important point. Last year we started the new Special Interest Group (SIG), which we call "Young World Federation for Neurorehabilitation" (Young WFNR). Elia Fischer from Berne, in Switzerland, is running this as the chairperson. And already now we have now roughly between 80–90 new SIG members from all over the world. From this came the idea to install a mentee-mentor program, in the sense that more experienced people can work as mentors in particular fields – there may be an expert in spasticity, there may be an expert in TBI, there may be an expert in stroke or whatever. And on the other hand, there may be mentees who would like to have a person to help them in their academic and clinical careers. The mentees can ask certain questions, and a person who could be helpful – as a mentor is, could be helpful to shape and marshal their careers, and is able to talk to them once a week or once a month, depending on needs, so that they are not lost in the field, but have some assistance in finding their way into a good academic and clinical career. That's the idea behind that. At the moment, we are just starting the first mentor-mentee matchings. Up to now, we have done about twelve matchings, with the number steadily increasing.

**A.G.:** Yes, because you implemented this mentorship program also at the level of the European Federation of NeuroRehabilitation.

**V.H.:** Yes, it's the same. We mirror this also in the European Federation, I didn't mention that before, but it is a good idea that the WFNR and the EFNR, the European Federation of NeuroRehabilitation, are working very closely together. We have, in almost every respect, parallel activities.

**A.G.:** Thank you very much, Professor Hömberg, for the interview, and we wish you the best of luck in your mandate!

**V.H.:** Thank you very much!

